# Predictors and Interdependence of Quality of Life in a Random Sample of Long‐Term Young Breast Cancer Survivors and Their Biological Relatives

**DOI:** 10.1002/cam4.70328

**Published:** 2024-10-29

**Authors:** Katrina R. Ellis, Helen Koechlin, Marion Rudaz, Lynette Hammond Gerido, Hillary K. Hecht, Carly Jones, Dolapo Raji, Laurel Northouse, Maria Katapodi

**Affiliations:** ^1^ School of Social Work University of Michigan Ann Arbor Michigan USA; ^2^ School of Public Health University of Michigan Ann Arbor Michigan USA; ^3^ Research Center for Group Dynamics Institute for Social Research, University of Michigan Ann Arbor Michigan USA; ^4^ University of Zurich Zürich Switzerland; ^5^ Department of Clinical Research University of Basel Basel Switzerland; ^6^ Case Western University Cleveland Ohio USA; ^7^ University of North Carolina at Chapel Hill Chapel Hill North Carolina USA; ^8^ Michigan Medicine University of Michigan Ann Arbor Michigan USA; ^9^ School of Nursing University of Michigan Ann Arbor Michigan USA

**Keywords:** family, interdependence, quality of life, young breast cancer survivors

## Abstract

**Purpose:**

Quality of life (QOL) among young breast cancer survivors (YBCS) is often worse than QOL of older breast cancer survivors or age‐matched peers without a history of cancer. Families commonly support YBCS, particularly during treatment, but little is known about long‐term YBCS and family member QOL. The purpose of this study was to identify demographic, clinical, and psychosocial predictors of physical and mental QOL in YBCS and biological relatives and investigate associations between their QOL (i.e., QOL interdependence).

**Methods:**

This secondary data analysis includes a random sample of long‐term YBCS (≤ 45 years old at diagnosis) and up to two female relatives at baseline (post‐treatment) and 18‐month follow‐up. The sample consists of 189 dyads (YBCS and one relative) and 121 triads (YBCS and two relatives). Actor‐partner interdependence models (APIMs) were used to estimate the influence of YBCS's and relatives' demographic, clinical, and psychosocial factors on their own QOL (actor effects) and the other persons' QOL (partner effects).

**Results:**

For YBCS and relatives, QOL at the baseline was associated with their QOL at 18‐months. YBCS's perceived cancer risk was associated with their own and relatives' QOL. Older relatives' physical QOL at baseline was associated with younger relatives' physical QOL at follow‐up. Age, race, marital status, years since diagnosis, education, out‐of‐pocket costs of care, routine sources of care, income, family support, fear of recurrence, anxiety, and depression were also significant predictors of QOL.

**Conclusions:**

Findings revealed independent and interdependent effects on QOL. These predictors point to potential targets of support for families.

**Trial Registration:**

ClinicalTrials.gov ID: NCT01612338

## 1 Introduction

Advances in screening and treatment have led to growing numbers of women diagnosed with breast cancer at younger ages. Approximately, 20%–25% of all new cases of breast cancer in the United States occur in women before age 45 [1, 2, 3]. These women, referred to here as young breast cancer survivors (YBCS), face specific health challenges, such as increased risk of a second breast cancer [4] and being diagnosed with a hereditary form of the disease [5]. Given that YBCS are living longer post‐diagnosis, it is important to consider implications for supportive care during an extended survivorship phase [6, 7].

Quality of life (QOL) is a key health outcome that warrants greater attention in this survivor population. Compared to older breast cancer survivors, YBCS exhibit greater deficits in various clinical and psychosocial factors that influence physical and mental QOL, such as greater levels of fatigue, increased occurrence of hot flashes, worse sleep quality, and persistent fear of cancer recurrence [8, 9, 10]. A study examining trends in health‐related QOL reported that 27% of YBCS met the diagnostic criteria for clinical depression compared to 17% of their age‐matched counterparts [9].

Although family members are often the primary source of support to help YBCS cope with the negative effects of cancer, family members' lives are also affected by the YBCS's illness [11]. The challenges reported by cancer survivors (e.g., emotional distress) can “spill‐over” to family members and negatively affect family members' quality of life [12]. Prior research has also found that family members often experience anxiety and depression, sleep disturbances, fatigue, and a lack of confidence in their supportive, caregiving roles [13].

Interdependence is an important concept to examine among YBCS and their family members and a cornerstone of this study. Interdependence has been described as “the process by which interacting people influence one another's experiences” (Van Lange et al., 2015, p. 65) [14]. Interdependence theory provides a framework for understanding the influence an individual has on their appraisals, emotions, behaviors and ultimately, their well‐being, and the well‐being of others with whom they interact (e.g., family members) [15, 16, 17, 18]. Moreover, interdependence should be examined with proper attention to key characteristics of the individuals and contexts where interactions occur. For example, within familial and caregiving contexts, individual characteristics (e.g., age and gender) and psychological orientations (e.g., perceptions of support) take on meanings that reflect intrapersonal, interpersonal, and broader social norms, values, and expectations. The examination and recognition of the influence individuals have on themselves (i.e., actor effects) as well as the influence they have on others (i.e., partner effects) can help to uncover key interdependent processes that can be targeted in supportive efforts.

In the field of cancer survivorship, several frameworks and studies call for approaches that recognize the interdependent responses of cancer survivors and their family members to the disease [12, 19, 20, 21]. As a whole, however, more research is needed to respond effectively to the documented and expanding needs of families [22, 23]. The family survivorship model [24] is one such framework that support a family‐focused orientation to cancer survivorship. In this model, family hardiness and social support are seen as key factors that influence the meaning derived from a cancer experience. Within this model and as supported by related literature (e.g., [25, 26, 27, 28]), relational factors such as perceptions of family communication, family support, and a family's ability to respond to challenges are worthwhile to consider when evaluating cancer survivors' and family members' ability to manage their health in the face of past, ongoing, and future health challenges.

The cancer family caregiving experience model also helps explain the state of family health and well‐being during cancer survivorship, including as main components contextual factors, the stress process, and the cancer trajectory [29]. Contextual factors in this model include socioeconomic, cultural, and health care characteristics, including social support and relationship quality. Characteristics such as age, gender, education, employment, and income are also highlighted in the model for their conceptual and practical importance. The stress process component of this model reflects aspects of the care recipient's illness, spillover effects of caregiving (e.g., changes in employment status), and cognitive‐behavioral responses (e.g., caregiving behaviors) of the caregiver. Taken together, this model helps identify mechanisms by which YBCS and family members may influence one another's mental and physical well‐being and worthwhile associations to investigate in ongoing health research.

Several other empirical studies also provide evidence of interdependent effects. Kershaw et al. [20] found that patients and their family caregivers influenced one another's mental and physical health but not their self‐efficacy. Dorros et al. [19] found that high levels of depression and stress in breast cancer patients was associated with lower physical health and well‐being in their partners. Furthermore, Litzelman et al. [12] found that cancer patients who had family caregivers with more depressive symptoms believed they received lower quality of care at home. In a systematic review of 103 studies, Meyler et al. [30] found evidence for concordant mental health, physical health, and health behaviors in couples coping with cancer, with the degree of concordance increasing as couples get older. In another systematic review, Streck et al. [31] found stronger interdependence between cancer patients' and their family caregivers' psychological morbidity compared to their physical morbidity.

Despite this evidence, critical questions remain about longer‐term survivor and family interdependence during the post‐treatment survivorship phase [31]. YBCS and family members can experience dramatic changes in relationships, roles, and psychological health when managing a cancer diagnosis, treatment, and post‐treatment transitions [32, 33]. Family caregivers, who may provide care to cancer survivors for years after the initial diagnosis and treatment, have reported negative impacts on their QOL and psychological distress [33]. Given that YBCS may be managing responsibilities and expectations related to parenting children and/or caregiving for elderly parents at the time of their cancer diagnosis, their family members may take on these additional responsibilities while they are also supporting the YBCS during survivorship. In addition, biological relatives of YBCS may have concerns about their own risk of breast cancer and the possibility of a genetic predisposition within the family. Family history has long been recognized among lay individuals as the foremost important risk factor for breast cancer [34]. These perceptions are well‐founded, given that biological relatives of YBCS have 1.5–2.3‐fold increased risk for the disease, depending on their degree of biological relation to the YBCS [2, 35, 36]. Thus, family support and open communication can provide opportunities to learn about cancer risk and risk reduction strategies [37].

To date, few studies examine the QOL of YBCS and their biological relatives interdependently after the acute phases of diagnosis and treatment and, specifically, several years after the end of cancer treatment. This information may be useful for designing health interventions for YBCS and relatives and for family‐based cancer survivorship initiatives, adding to the critical, ongoing broader research on improving QOL across the cancer continuum [38, 39, 40]. Thus, the purpose of this study was to examine factors that influence QOL of long‐term YBCS and their female, first‐ or second‐degree biological relatives. Two research questions guided this study. First, are demographic, clinical (years since diagnosis, history of anxiety, and depression), and psychosocial characteristics (fear of recurrence, perceived breast cancer risk, family support) associated with the QOL of YBCS and their biological relatives? Considering demographic characteristics, in addition to clinical and psychosocial factors, is helpful for increasing understanding of the individual and interpersonal factors that influence well‐being [31]. Second, is there interdependence between the QOL of YBCS and their relatives during the long‐term, post‐treatment phase of cancer survivorship? Research that takes into account the complexities of family relationships (and associated theories) and includes the analysis of robust data from multiple family members could increase understanding of factors influencing cancer survivorship and quality of life among family units.

## 2 Method

### 2.1 Design and Sample

This is a secondary analysis of baseline and 18‐month follow‐up data from a randomized controlled trial (RCT) designed to increase genetic awareness among YBCS and first‐ and second‐degree female relatives (NCT01612338) [41]. Institutional Review Board approval and participant informed consent were obtained. Details regarding study design, recruitment, and findings have been reported elsewhere [41, 42, 43]. In brief, a random sample of 3000 YBCS was obtained from the Michigan Cancer Surveillance Program (state cancer registry) and YBCS were invited to the RCT. The sample was stratified by race (1500 Black/African American YBCS and 1500 White/Other YBCS) to ensure adequate representation of Black/African American YBCS. The “Other” category included approximately 6% of YBCS not recorded in the cancer registry as Black/African American or White, for example, Arab Americans, who were grouped with White YBCS. YBCS were eligible to participate if they were female; diagnosed with invasive breast cancer or ductal carcinoma in situ (DCIS); younger than 45 years at the time of diagnosis and 25–64 at the time of the study; Michigan residents at the time of diagnosis; not pregnant or institutionalized during recruitment; and willing to invite one or two relatives to the study. Relatives were eligible to participate if they were female; first‐ or second‐degree relatives of the YBCS; 25–64 years old; and, cancer‐free at the time of recruitment. The study invited up to two relatives per YBCS to have family units of comparable sizes. Participants received $10 and $20 gift cards at baseline and at follow‐up, respectively, as compensation for their participation in the study. Baseline data for the RCT were collected prior to the intervention trial; follow‐up data were collected 18‐months after the trial.

Of the 3000, YBCS invited to participate, 883 agreed to join the study (response rate: 33.2% after excluding deceased, YBCS with missing contact information, and individuals not meeting eligibility criteria). Among these, there were 573 YBCS who participated in the RCT alone (i.e., without biological relatives) and were excluded from the present analysis. YBCS participated alone for various reasons, including not having eligible relatives, not having relatives willing to participate, or not willing to contact relatives. Black YBCS were less willing to invite older relatives (*p* = 0.001) and relatives living more than 50 miles away (*p* = 0.027) to the study compared to White YBCS. The final sample of the current analysis includes 189 family units with a YBCS and one relative (i.e., dyads) and 121 family units with a YBCS and two relatives (i.e., triads). The majority (84.2%) of enrolled relatives consited of first‐degree relatives (i.e., sisters, daughters, and mothers; 84.2%). The specific relatives enrolled included 231 sisters (53.6%), 123 daughters (28.5%), 41 nieces (9.5%), 14 half‐sisters (3.2%), 13 aunts (3.0%), and 9 mothers (2.1%). Most of the enrolled relatives (*n* = 313, 72.6%) resided in Michigan.

### 2.2 Measurement

#### 2.2.1 Quality of Life

The physical and mental QOL of YBCS and relatives (i.e., dependent variables) were assessed at baseline and 18‐month follow‐up with the Short‐Form Health Survey (SF‐12) [44]. SF‐12 is an established instrument that assesses health‐related QOL with 12 items that address eight domains: (1) physical functioning (two questions asking about limitations due to health); (2) physical role function (two questions asking if physical health leads to problems with work and other daily activities); (3) bodily pain (one question asking about interference of pain with daily life); (4) general health perception (one question asking about general perception of one's health); (5) vitality (one question asking about having energy); (6) social functioning (one question asking about interference of physical or emotional problems with social life); (7) emotional role function (two questions asking about problems with daily life as a result of emotional problems); and (8) mental health (two questions asking about feeling blue or peaceful). Individual domain scores are used to create a physical and a mental QOL composite score. The physical and mental QOL scores range from 0 to 100, with higher scores indicating better QOL. Internal consistency of the 12 items was good for YBCS (*α* = 0.895) and relatives (*α* = 0.854). When separately evaluating the items most closely related to the two subscales [45, 46], the internal consistency was also good for physical QOL (YBCS: *α* = 0.876; relatives: *α* = 0.825) and mental QOL (YBCS: *α* = 0.838; relatives: *α* = 0.794), specifically.

#### 2.2.2 Demographic Characteristics

Age, YBCS race category (Black/African American vs. White/Other), education, income, employment (full‐time vs. part‐time, unemployed, or other), and marital status (married or living as married vs. single, divorced, or widowed) were assessed at baseline with items from the Behavioral Risk Factors Surveillance System Survey [47]. Cost‐related barriers to accessing care for YBCS and relatives were assessed with a yes/no question: “Has there been a time within the past 12 months that you needed to see a doctor or have a medical test but you could not because of high out‐of‐pocket cost?” Access to a routine source of healthcare was assessed with a yes/no question: “Do you receive your annual checkups and prevention services from the same office (or offices), such as a primary care or OB/GYN office?”

#### 2.2.3 Clinical Characteristics

Years since YBCS cancer diagnosis was assessed at baseline and calculated as a continuous variable. Previous diagnoses of anxiety and depression were assessed at baseline with two questions, which asked if participants had ever been told by a healthcare provider that they had anxiety or depression (yes/no).

#### 2.2.4 Psychosocial Characteristics

Fear of recurrence was assessed at baseline among YBCS with four items from the Concerns About Recurrence Scale (CARS) using a seven‐point Likert scale ranging from “Not at all” to “All the time” [48]. The questions address the amount of time, frequency, intensity, and potential for upset or fear related to cancer recurrence. Internal consistency was 0.91 in this sample.

Perceived breast cancer risk was assessed at baseline among YBCS and relatives with a single item asking them to estimate the chances of developing (another) breast cancer on a 10‐point Likert scale with verbal anchors ranging from “Definitely will not” to “Definitely will” [49].

A family support index was created from three scales measuring different attributes of family support, which were assessed given at baseline to YBCS and relatives [5]. The Lewis Mutuality and Interpersonal Sensitivity Scale (MIS) [50] measures levels of open family communication. It consists of 15 items, such as “The people in my family change the topic when I discuss my concerns”. The Family Support in Illness [51] assesses family support specifically at times of illness. It consists of 10 items, such as “In our family, when I have a health problem, there is someone helping me get the care that I need”. The Family Hardiness Index [52] measures family coherence, that is, a family's ability to cope with adverse events. It consists of 20 items, such as “In our family, we have a sense of being strong even when we face big problems”. All items were rated on a 7‐point Likert scale from 1 (strongly disagree) to 7 (strongly agree). Principal component analysis led to the identification of a primary component of family support; four items were not used because they did not correlate adequately with the principal component. Internal consistency for the retained items was good for YBCS (*α* = 0.966) and relatives (*α* = 0.963). We calculated a mean score from the three scales as an overall family support index score, with higher scores indicating higher levels of family support [5].

### 2.3 Statistical Analyses

Sample characteristics were described with means, standard deviations (SD), frequencies (*N*), or percentages (%), depending on scaling and data distribution. Data from YBCS and relatives are not assumed to be independent given the interpersonal relationships that existed. Thus, correlations between their data were factored into analytic techniques [53]. The Actor‐Partner Interdependence Model (APIM) is useful for examining interdependence and bidirectional effects within interpersonal contexts [54]. Use of the APIM with dyads requires examining the dyad (i.e., YBCS and relative) as the unit of analysis instead of as distinct individuals. Extending the APIM to triads (i.e., YBCS and two relatives) requires the same consideration: the YBCS, an older and a younger relative comprising the unit of analysis (i.e., relatives distinguished by age). Actor effects among YBCS and relatives are observed when an individual's own characteristics are predictive of their own outcome (e.g., YBCS's depression predictive of YBCS's mental QOL). Partner effects are observed when an individual's characteristics are predictive of the outcomes of another individual in the dyad or triad (e.g., YBCS's depression predictive of relatives' mental QOL).

**FIGURE 1:**
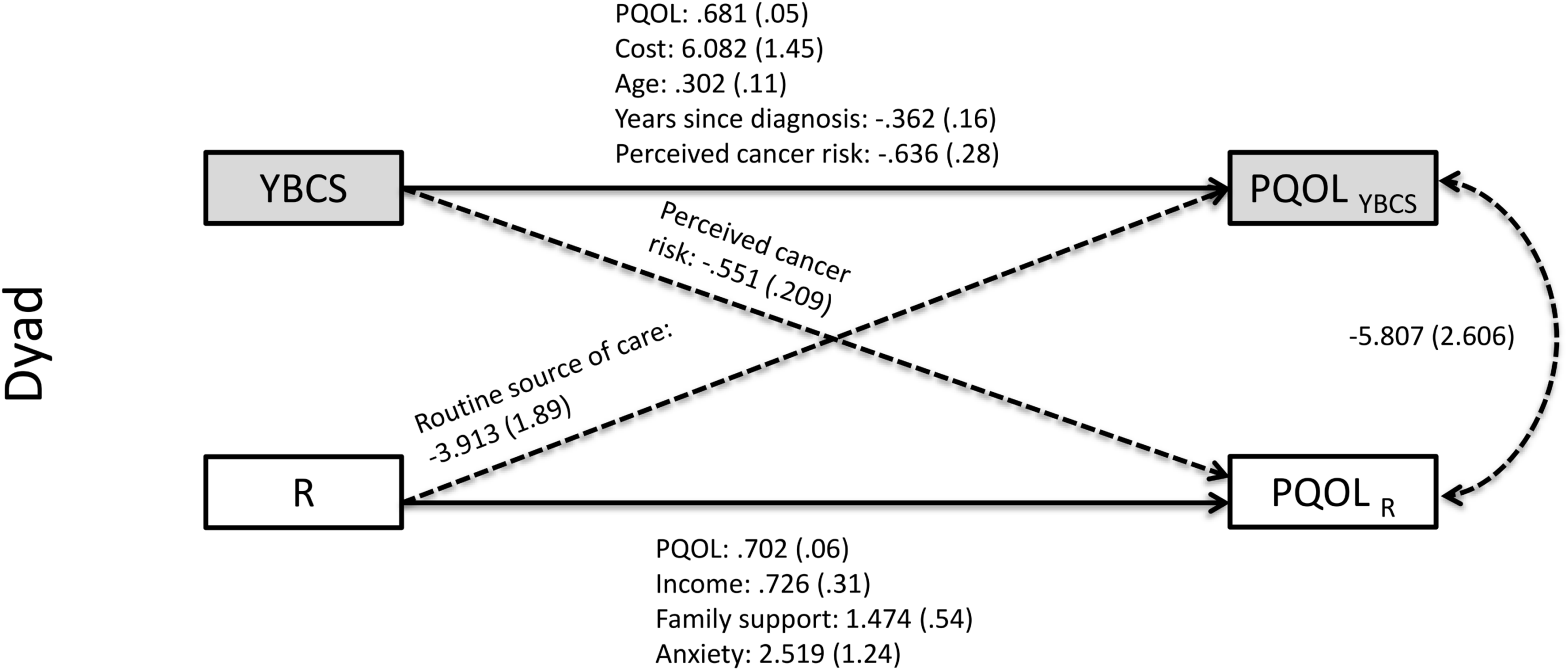
Dyad—significant predictors of physical quality of life. Actor–Partner Interdependence Model (APIM) of quality of life in YBCS and their biological relatives (dyads). Solid lines: actor effects. Dashed lines: partner effects. Statistically significant (*p* < 0.05) unstandardized coefficients for paths are displayed, followed by standard errors in parentheses. Model fit indices (dyad‐physical): Chi‐square (*p* = 0.665); *X* ^2^/df ratio = 0.786; RMSEA = 0.000; and SRMR = 0.005. PQOL, physical quality of life; R, relative; YBCS, young breast cancer survivor.

**FIGURE 2:**
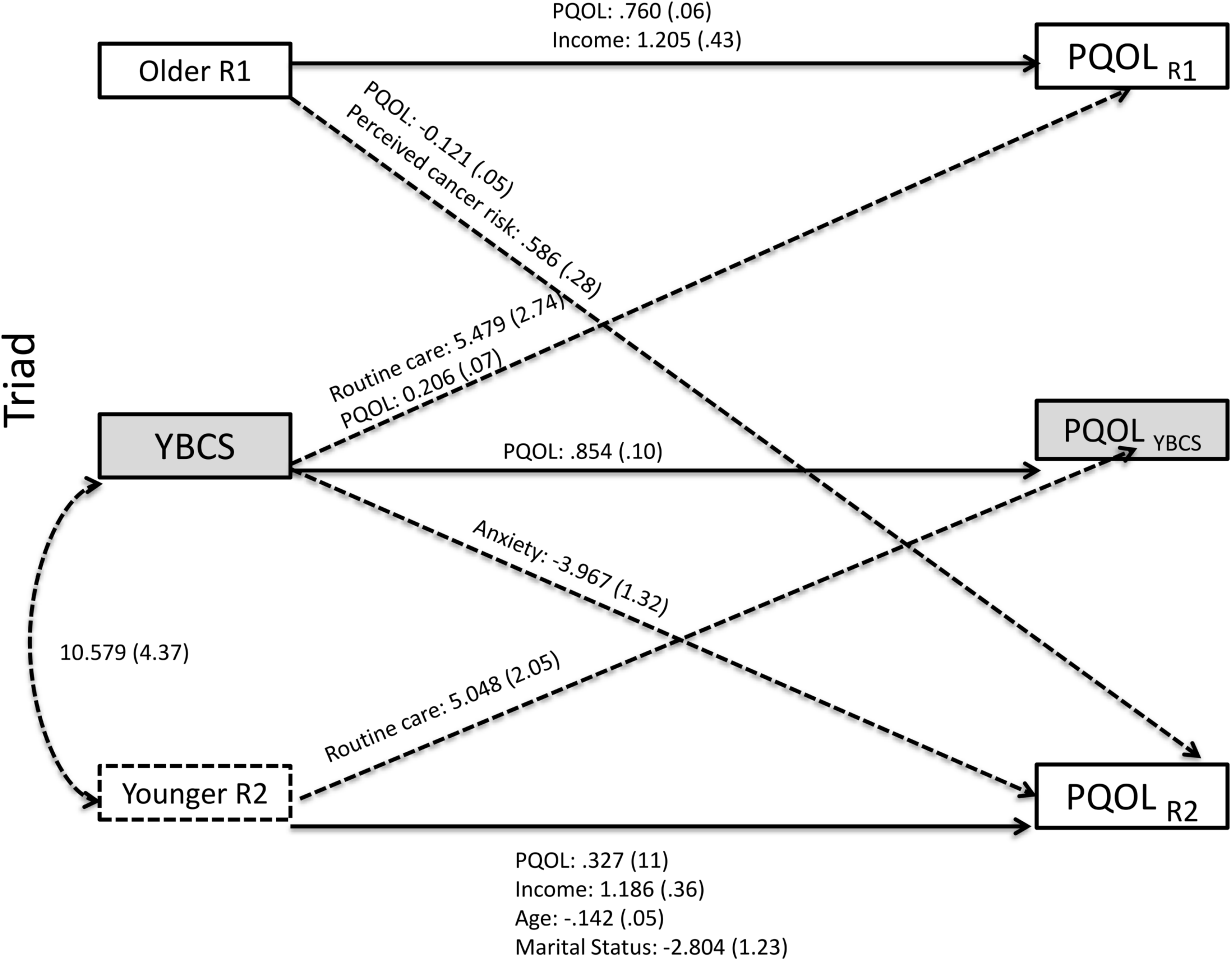
Triad—significant predictors of physical quality of life. Actor–Partner Interdependence Model (APIM) of quality of life in YBCS and their older and younger biological relatives (triads). Solid lines: Actor effects. Dashed lines: Partner effects. Statistically significant (*p* < 0.05) unstandardized coefficients for paths are displayed, followed by standard errors in parentheses. Model fit indices (triad‐physical): Chi‐square (*p* = 0.121); *X* ^2^/df ratio = 1.31; RMSEA = 0.050; and SRMR = 0.014. PQOL, physical quality of life; R, relative; YBCS, young breast cancer survivor.

**FIGURE 3:**
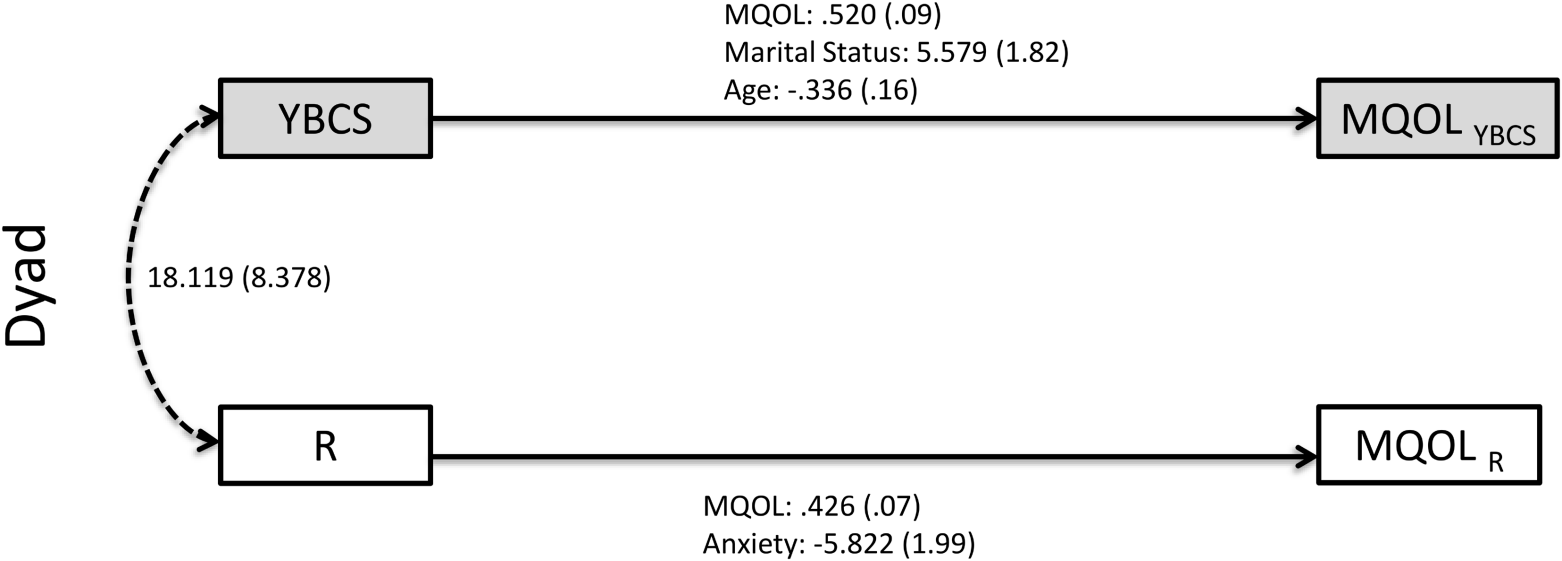
Dyad—significant predictors of mental quality of life. Actor–Partner Interdependence Model (APIM) of quality of life in YBCS and their biological relatives (dyads). Solid lines: actor effects. Dashed lines: partner effects. Statistically significant (*p* < 0.05) unstandardized coefficients for paths are displayed, followed by standard errors in parentheses. Model fit indices (dyad‐mental): Chi‐square (*p* = 0.722); *X* ^2^/df ratio = 0.585; RMSEA = 0.000; and SRMR = 0.007. MQOL, mental quality of life; R, relative; YBCS, young breast cancer survivor.

**FIGURE 4:**
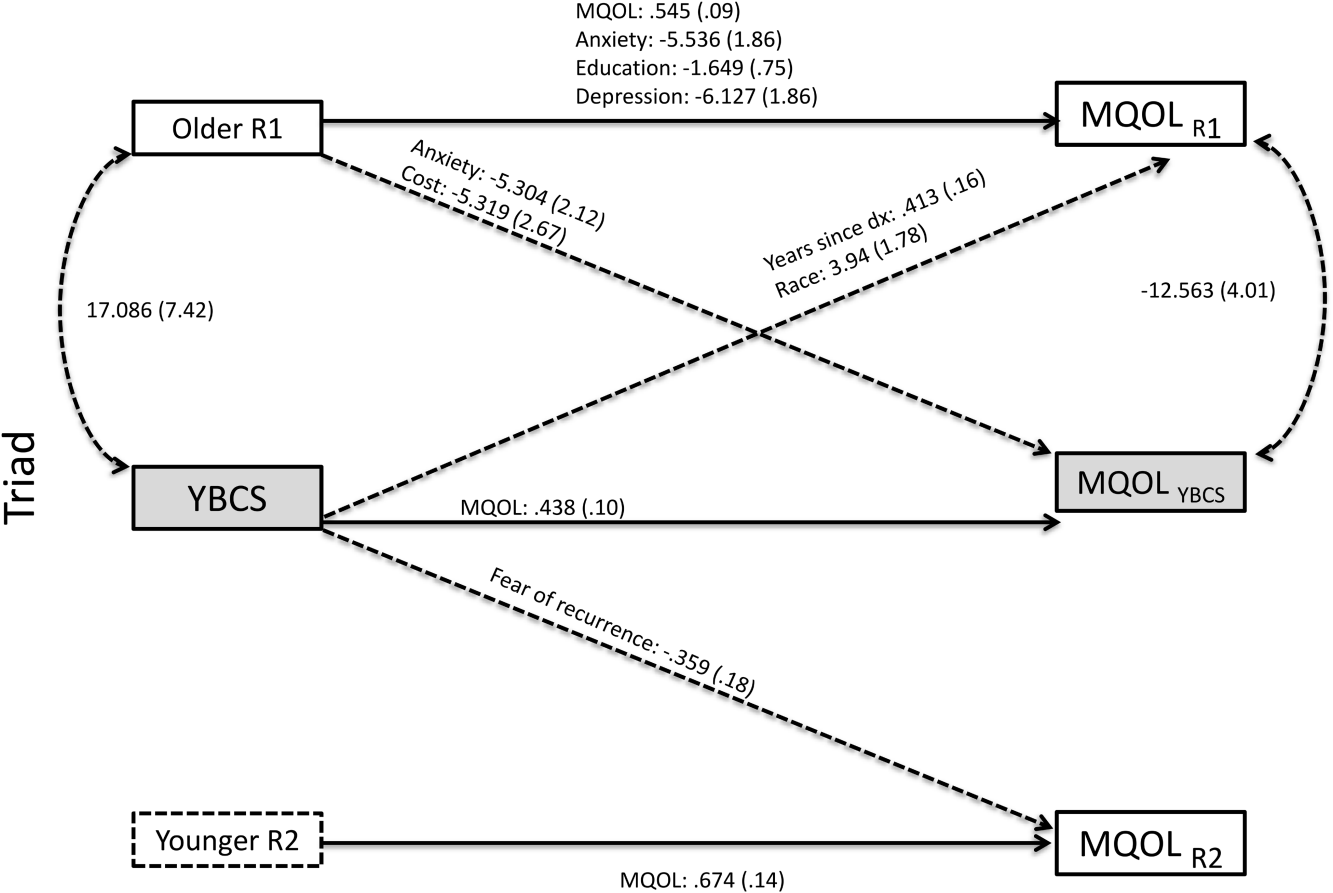
Triad—significant redictors of mental quality of life. Actor–Partner Interdependence Model (APIM) of quality of life in YBCS and their older and younger biological relatives (triads). Solid lines: actor effects. Dashed lines: partner effects. Statistically significant (*p* < 0.05) unstandardized coefficients for paths are displayed, followed by standard errors in parentheses. Model fit indices (triad‐mental): Chi‐square (*p* = 0.593); *X* ^2^/df ratio = 0.919; RMSEA = 0.000; and SRMR = 0.010. MQOL, mental quality of life; R, relative; YBCS, young breast cancer survivor.

Data were structured in dyadic and triadic formats, such that each observation included data from a full dyad or full triad. Full information maximum likelihood estimation (FIML) was used in path analyses predicting YBCS and relative QOL; intervention group (stemming from the original efficacy trial) was controlled for in the analysis by including an intervention group assignment variable as a control variable. Several indicators of adequate model fit were evaluated: a non‐significant chi‐square statistic or a ratio of chi‐square statistic to degrees of freedom (*df*) less than two, a root mean squared error of approximation (RMSEA) value ≤ 0.08, and a standardized root mean square residual (SRMR) value ≤ 0.08 [55, 56]. Model fit was acceptable (see notes on Figures 1, 2, 3, 4). Descriptive analyses were performed with SPSS version 27.0 [57], and APIM analyses were performed with MPlus version 8.0 [58].

## 3 Results

### 3.1 Sample Characteristics

#### 3.1.1 Dyads

At baseline, YBCS were significantly older than relatives in the dyadic sample (mean ages 51.7 vs. 43.8 years old; Table 1). Although relatives reported significantly higher levels of education compared to YBCS, there were no other differences between YBCS's and relatives' demographic, clinical, and psychosocial characteristics. Although YBCS reported significantly lower physical QOL compared to relatives (mean score 48.7 vs. 51.5), there was no difference between the average mental QOL scores of YBCS and relatives (mean score 48.7 vs. 49.2).

**TABLE 1:** Dyad sample characteristics.

	YBCS (*N* = 189)	Relatives (*N* = 189)	Difference tests (*p*)
Demographics			
Age (mean ± SD)	51.7 ± 5.6	43.8 ± 11.8	< 0.001
Race (*n*, %)			0.125
Black/African American	44 (23.3)	39 (20.6)	
White and other	145 (76.7)	150 (79.4)	
Education (*n*, %)			< 0.001
Elementary school only	—	1 (0.5)	
Some high school	1 (0.5)	7 (3.8)	
High school graduate	36 (19.3)	22 (11.8)	
Some college	69 (36.9)	64 (34.4)	
College graduate	40 (21.4)	56 (30.1)	
Postgraduate degree	41 (21.9)	36 (19.4)	
Employment (*n*, %)			0.189
Full time	88 (49.7)	105 (56.8)	
Part‐time/unemployed/other	89 (50.3)	80 (43.2)	
Income (*n*, %)			0.081
< $20,000	17 (10.1)	21 (12.2)	
$20,000–$39,999	30 (17.8)	37 (21.5)	
$40,000–$59,999	28 (16.6)	28 (16.3)	
$60,000–$79,999	25 (14.8)	23 (13.4)	
$80,000–$99,999	20 (11.8)	19 (11.0)	
$100,000–$119,999	20 (11.8)	16 (9.3)	
> $120,000	29 (17.2)	28 (16.3)	
Marital status (*n*, %)			0.119
Married/living as married	123 (65.1)	107 (56.9)	
Single/divorced/widowed	66 (34.9)	81 (43.1)	
No out‐of‐pocket cost barriers to care (*n*, %)	150 (79.4)	149 (80.1)	1.00
Routine source of care	176 (93.1)	168 (89.8)	0.230
Clinical characteristics			
Years since 1st diagnosis (mean ± SD)	11.7 ± 4.0	—	—
Anxiety (*n*, %)	62 (33.2)	45 (24.6)	0.092
Depression (*n*, %)	55 (29.7)	50 (27.3)	1.00
Psychosocial characteristics			
Fear of recurrence	13.8 ± 6.7	—	—
Perceived breast cancer risk	5.3 ± 2.4	4.6 ± 2.1	0.006
Family support (mean ± SD)	5.6 ± 1.0	5.6 ± 0.9	0.509
Quality of life (SF‐12)			
Physical (mean ± SD)	48.7 ± 10.7	51.5 ± 8.8	0.002
Mental (mean ± SD)	48.7 ± 10.4	49.2 ± 10.9	0.834

Abbreviation: YBCS, young breast cancer survivors.

#### 3.1.2 Triads

At baseline, YBCS and older relatives were each significantly older than younger relatives (mean ages 50.9 vs. 48.9 vs. 37.1 years old; Table 2). YBCS reported significantly higher levels of income compared to younger relatives and were more likely than older relatives to be married/living as married. Younger relatives reported significantly higher levels of education and were more likely to be employed full time compared to older relatives but not compared to YBCS. There were no other differences between YBCS's and relatives' demographic, clinical, and psychosocial characteristics. YBCS's physical QOL was similar to that of older relatives (mean score 50.9 vs. 49.2) but significantly lower compared to younger relatives (mean score 50.9 vs. 53.9). There were no differences between the mental QOL scores of YBCS, older relatives, and younger relatives (mean scores 51.1 vs. 51.9 vs. 51.0).

**TABLE 2:** Triad sample characteristics.

	YBCS (*N* = 121)	Older relatives (*N* = 121)	Younger relatives (*N* = 121)	Difference tests (*p*)		
				YBCS vs. Older relatives	YBCS vs. Younger relatives	Older vs. Younger relatives
Demographics						
Age (mean ± SD)	50.9 ± 6.1	48.9 ± 10.2	37.1 ± 10.5	0.081	< 0.001	< 0.001
Race (*n*, %)				1.00	1.00	1.00
Black/African American	24 (19.8)	24 (19.8)	24 (19.8)			
White and other	97 (80.2)	97 (80.2)	97 (80.2)			
Education (*n*, %)				0.101	0.562	0.008
Elementary school only	—	—	1 (0.8)			
Some high school	1 (0.8)	2 (1.7)	—			
High school graduate	20 (16.8)	27 (22.9)	12 (10.0)			
Some college	44 (37.0)	46 (39.0)	47 (39.2)			
College graduate	34 (28.6)	24 (20.3)	46 (38.3)			
Postgraduate degree	20 (16.8)	19 (16.1)	14 (11.7)			
Employment (*n*, %)				0.341	0.576	0.026
Full time	69 (59.0)	58 (50.9)	74 (63.2)			
Part‐time/unemployed/other	48 (41.0)	56 (49.1)	43 (36.8)			
Income (*n*, %)				0.300	0.003	0.517
< $20,000	7 (6.5)	17 (16.7)	13 (11.6)			
$20,000–$39,999	12 (11.1)	21 (20.6)	26 (23.2)			
$40,000–$59,999	22 (20.4)	16 (15.7)	24 (21.4)			
$60,000–$79,999	26 (24.1)	14 (13.7)	16 (14.3)			
$80,000–$99,999	10 (9.3)	9 (8.8)	8 (7.1)			
$100,000–$119,999	9 (8.3)	9 (8.8)	8 (7.1)			
> $120,000	22 (20.4)	16 (15.7)	17 (15.2)			
Marital status (*n*, %)				0.036	0.098	0.878
Married/living as married	89 (73.6)	74 (61.2)	76 (62.8)			
Single/divorced/widowed	32 (26.4)	47 (38.8)	45 (37.2)			
No out‐of‐pocket cost barriers to care (*n*, %)	104 (86.0)	101 (83.5)	96 (79.3)	0.720	0.243	0.424
Routine source of care	115 (95.0)	112 (93.3)	100 (84.0)	0.774	0.015	0.017
Clinical characteristics						
Years since 1st diagnosis (mean ± SD)	11.3 ± 3.9	—	—	—	—	—
Anxiety (*n*, %)	27 (22.7)	32 (26.4)	38 (31.4)	0.736	0.132	0.480
Depression (*n*, %)	29 (24.2)	28 (23.1)	33 (27.3)	0.871	0.728	0.542
Psychosocial characteristics						
Fear of recurrence	13.3 ± 5.8	—	—	—	—	—
Perceived breast cancer risk	4.6 ± 1.8	4.5 ± 1.8	4.8 ± 2.0	0.611	0.447	0.187
Family support (mean ± SD)	5.7 ± 0.8	5.8 ± 0.8	5.8 ± 0.8	0.236	0.130	0.775
Quality of life (SF‐12)						
Physical (mean ± SD)	50.9 ± 8.5	49.2 ± 10.5	53.9 ± 5.4	0.255	< 0.001	< 0.001
Mental (mean ± SD)	51.1 ± 9.5	51.9 ± 8.0	51.0 ± 8.3	0.716	0.801	0.659

Abbreviation: YBCS, young breast cancer survivors.

### 3.2 Predictors of Physical QOL

#### 3.2.1 Dyads

APIM analyses identified several actor and partner effects for physical QOL in dyads (see Tables 3 and 4; Figure 1 and Tables S1 and S2). With regards to actor effects, among YBCS, age, physical QOL, and lack of cost‐related barriers to healthcare at baseline were positive predictors of their own physical QOL at follow‐up. In contrast, years since their cancer diagnosis and their perceived breast cancer risk at baseline were negative predictors of YBCS's own physical QOL at follow‐up. Among relatives, their physical QOL, income, history of anxiety, and family support at baseline were positive predictors of their own physical QOL at follow‐up.

**TABLE 3:** YBCS factors predicting physical and mental quality of life of YBCS and relatives.

	Dyad		Triad	
	B (SE)	*p*	B (SE)	*p*
Actor effects				
Physical quality of life—Actor effects				
YBCS age ➔ YBCS physical QOL	0.302 (0.11)	0.008		
YBCS no out‐of‐pocket cost barriers to care ➔ YBCS physical QOL	6.082 (1.45)	< 0.001		
YBCS years since diagnosis ➔ YBCS physical QOL	−0.362 (0.16)	0.022		
YBCS perceived breast cancer risk ➔ YBCS physical QOL	−0.636 (0.28)	0.005		
YBCS physical QOL ➔ YBCS physical QOL	0.681 (0.05)	< 0.001	0.854 (0.10)	< 0.001
Partner effects				
Physical quality of life—Partner effects				
YBCS perceived breast cancer risk ➔ Relative physical QOL	−0.551 (0.21)	0.009		
YBCS routine source of care ➔ Older relative physical QOL			5.479 (2.74)	0.045
YBCS anxiety ➔ Younger relative physical QOL			−3.967 (1.32)	0.003
YBCS physical QOL ➔ Older relative physical QOL			0.206 (0.07)	0.007
Actor effects				
Mental quality of life—Actor effects				
YBCS age	−0.336 (0.16)	0.037		
YBCS marital status	5.579 (1.82)	0.002		
YBCS mental QOL ➔ YBCS mental QOL	0.520 (0.09)	< 0.001	0.438 (0.10)	< 0.001
Partner effects				
Mental quality of life—Partner effects				
YBCS years since diagnosis➔ Older relative mental QOL			0.413 (0.16)	0.011
YBCS race ➔ Older relative mental QOL			3.94 (1.78)	0.027
YBCS fear of recurrence ➔ Younger relative mental QOL			−0.359 (0.18)	0.043

*Abbreviations:* YBCS, young breast cancer survivors; QOL, quality of life.

**TABLE 4:** Relative factors predicting physical and mental quality of life of relatives and YBCS.

	Dyad		Triad			
	Relative		Older relative		Younger relative	
	B (SE)	*p*	B (SE)	*p*	B (SE)	*p*
Actor effects						
Physical quality of life—Actor effects						
Relative age ➔ Relative physical QOL					−0.142 (0.05)	0.005
Relative income ➔ Relative physical QOL	0.726 (0.31)	0.020	1.205 (0.43)	0.017	1.186 (0.36)	0.001
Relative marital status ➔ Relative physical QOL					−2.804 (1.23)	0.022
Relative anxiety ➔ Relative physical QOL	2.519 (1.24)	0.042				
Relative family support ➔ Relative physical QOL	1.474 (0.54)	0.007				
Relative physical QOL ➔ Relative physical QOL	0.702 (0.06)	< 0.001	0.760 (0.06)	< 0.001	0.327 (0.11)	0.004
Partner effects						
Physical quality of life—Partner effects						
Relative routine source care ➔ YBCS physical QOL	−3.913 (1.89)	0.036				
Older relative physical QOL ➔ Younger relative physical QOL			−0.121 (0.05)	0.026		
Older relative perceived breast cancer risk ➔ Younger relative physical QOL			0.586 (0.28)	0.038		
Younger relative routine source care ➔ YBCS physical QOL					5.048 (2.05)	0.014
Actor effects						
Mental quality of life—Actor effects						
Relative education ➔ Relative mental QOL			−1.649 (0.75)	0.028		
Relative anxiety ➔ Relative mental QOL	−5.822 (1.99)	0.003	−5.536 (1.86)	0.003		
Relative depression ➔ Relative mental QOL			−6.127 (1.86)	0.001		
Relative mental QOL ➔ Relative mental QOL	0.426 (0.07)	< 0.001	0.545 (0.09)	< 0.001	0.674 (0.14)	< 0.001
Partner effects						
Mental quality of life—Partner effects						
Older relative anxiety ➔ YBCS mental QOL			−5.304 (2.12)	0.012		
Older relative no out‐of‐pocket cost barriers to care ➔ YBCS mental QOL			−5.319 (2.67)	0.046		

Abbreviations: QOL, quality of life; YBCS, young breast cancer survivors.

With regards to partner effects in dyads, YBCS's perceived breast cancer risk at baseline was negatively associated with relatives' physical QOL at follow‐up (Table 3). Relatives having access to a routine source of healthcare was negatively associated with YBCS's physical QOL at follow‐up (Table 4).

#### 3.2.2 Triads

APIM analyses identified several actor and partner effects for physical QOL in triads (see Tables 3 and 4; Figure 2 and Tables S3–S5). With regards to actor effects, among YBCS, baseline physical QOL was positively associated with their own physical QOL at follow‐up. Actor effects were also identified among relatives. Older relatives' and younger relatives' physical QOL and income at baseline were positive predictors of their own physical QOL at follow‐up. In contrast, younger relatives' age and being married/living as married at baseline were negative predictors of their own physical QOL at follow‐up.

With regards to partner effects in triads, YBCS having access to a routine source of healthcare at baseline, and YBCS physical QOL at baseline, were positively associated with the physical QOL of older relatives at follow‐up. YBCS's history of anxiety at baseline was negatively associated with the physical QOL of the younger relatives at follow‐up. Older relatives' physical QOL at baseline was negatively associated with younger relatives' physical QOL at follow‐up. In contrast, older relatives' perceived breast cancer risk at baseline was positively associated with younger relatives' physical QOL at follow‐up. Finally, younger relatives having access to a routine source of healthcare at baseline was positively associated with YBCS's physical QOL at follow‐up.

### 3.3 Predictors of Mental QOL

#### 3.3.1 Dyads

APIM analyses identified several actors effects for mental QOL in dyads (see Tables 3 and 4; Figure 3 and Tables S6 and S7). Among YBCS (Table 3), mental QOL and being married/living as married at baseline were positive predictors of their own QOL at follow‐up. YBCS's age at baseline was a negative predictor of their own mental QOL at follow‐up. Among relatives (Table 4), mental QOL at baseline was a positive predictor of their own mental QOL at follow‐up, while history of anxiety at baseline was a negative predictor of their own mental QOL at follow‐up. APIM analyses did not identify any partner effects for mental QOL in the dyads.

#### 3.3.2 Triads

APIM analyses identified several actor and partner effects for mental QOL in triads (see Tables 3 and 4; Figure 4 and Tables S8–S10). With regards to actor effects, among YBCS, mental QOL at baseline was a positive predictor of their own mental QOL at follow‐up; similar findings were observed among older relatives and younger relatives. In contrast, older relatives' education, history of anxiety, and history of depression were negatively associated with their own mental QOL at follow‐up.

With regards to partner effects in triads, years since the YBCS's cancer diagnosis at baseline was positively associated with older relatives' mental QOL at follow‐up, indicating that a longer time since YBCS's diagnosis predicted better mental QOL in older relatives. Black racial identification of YBCS was also associated with better mental QOL among older relatives. In contrast, YBCS's fear of cancer recurrence at baseline was negatively associated with younger relatives' mental QOL at follow‐up. Moreover, lack of cost‐related barriers to healthcare among older relatives at baseline was negatively associated with YBCS's mental QOL at follow‐up. History of anxiety among older relatives at baseline was also negatively associated with YBCS's mental QOL at follow‐up, suggesting that anxiety among older relatives predicted worse mental QOL of YBCS.

## 4 Discussion

As the population of YBCS continues to grow [59, 60], it is important to understand factors that influence YBCS's well‐being as they progress into middle‐ and older‐age. Likewise, the exponential use of cancer genetics in clinical practice guidelines [61] spurs curiosity about how the QOL of biological relatives is impacted by the young age of cancer onset. This study examined how demographic, clinical, and psychosocial characteristics influence the long‐term QOL of YBCS and relatives as well as the potential interdependence among dyad and triad family units. YBCS's and relatives' QOL at baseline were positively associated with their own QOL at follow‐up (actor effects). This consistency across time is often seen in other studies of QOL among cancer survivors and family caregivers [20], suggesting that those with higher QOL continue to have better QOL over time. In addition, several predictors of QOL were observed for YBCS and relatives across dyad and/or triad models.

Time since the YBCS's cancer diagnosis seems to have a different impact on YBCS QOL compared to relative QOL. Years since diagnosis was negatively associated with YBCS's physical QOL (dyad model) but was positively associated with older relative mental QOL (triad model). It is generally understood that chemotherapy accelerates the aging process [62]; thus, it can be speculated that YBCS who were treated with chemotherapeutical agents have worse physical QOL due to accelerated age‐related physical functioning. YBCS may also experience regrets related to family planning or health challenges as they age stemming from cancer risk reduction and/or treatment strategies [63, 64].

With regards to the finding that more time since diagnosis is related to better relative mental QOL, it is possible that the longer YBCS have been in remission, the less their relatives are concerned about YBCS's overall well‐being. Data also indicate, however, that the passage of time alone may not be enough to protect relatives' well‐being. In the triad analysis, as YBCS's fear of recurrence increased, the mental QOL of life of younger relatives decreased. This finding of a partner effect (i.e., YBCS fear influencing relative QOL) adds to other research that found actor effects, whereby relatives' own fear of recurrence negatively influenced their well‐being. For example, APIM analysis of data collected 2 years after the initial cancer diagnosis from 455 cancer survivor–caregiver dyads showed that cancer severity was positively associated with fear of cancer recurrence among family caregivers, which was negatively associated with their mental health [65]. Other studies also reported that fear of cancer recurrence among family caregivers had a negative impact on their QOL [66, 67]. Taken together, evidence of actor and partner effects of fear of recurrence on well‐being supports addressing this psychosocial challenge among survivors and family members.

Higher income among relatives was positively associated with their own physical QOL in the dyadic and the triadic family units. Higher income is consistently linked to better health outcomes [68, 69], including among cancer survivors [70]. Reasons for this association include increased access to resources and reduced stress due to the privileges afforded by financial stability, such as more control over choices and decisions that affect one's life and livelihood [68, 71]. Considering this, it is interesting that income was not associated with physical or mental QOL of YBCS, since it is well documented that financial burden associated with cancer care has significant negative effects on physical and mental QOL among US cancer survivors [72, 73]. One possible explanation is that YBCS in our sample were on average 11 years post‐diagnosis, where there is often less need for intensive use of healthcare services compared to the time frame around cancer diagnosis and treatment. Moreover, more than 80% of participants reported that they did not face difficulties accessing healthcare services due to out of pocket expenses.

Anxiety among YBCS and relatives was associated with poorer physical and/or mental QOL in relatives. This finding is in line with previous studies that have observed connections between anxiety and well‐being among people with different types of cancer and their family members/caregivers. However, many of these studies occurred during or near the acute treatment phase [20, 65, 74], while less is known about the long‐term survivorship phase. The reverse mechanism also seems to be true as low QOL of cancer patients has been found to be an important predictor of anxiety among family caregivers [75].

Increased perceived breast cancer risk among YBCS was associated with poorer physical QOL among YBCS and relatives (dyad model). Other studies have also described that increased perception of breast cancer risk has a negative influence on QOL [76], while interdependence exists between patients' perceived levels of stress and caregivers' QOL [77]. An unexpected finding was that increased perceived breast cancer risk among older relatives was positively associated with physical QOL among younger relatives in the triad model. It is possible that younger relatives are more vigilant with their health practices, given that they have witnessed the cancer diagnosis of the YBCS and/or aging‐related health concerns of older relatives; previous studies link vigilance about one's health to individual experiences with anxiety [78, 79, 80]. Another possible explanation involves the mediating role of family communication [27]. In the context of this study, it is possible that older relatives sharing their perceptions of increased breast cancer risk with younger relatives may have motivated the latter to be more attentive to and vigilant about their physical QOL.

### 4.1 Interdependence, Caregiving, and QOL

Partner effects in dyad and triad models indicate interdependence in physical and mental health outcomes, highlighting various ways in which YBCS's characteristics influence relatives' QOL and vice versa [81, 82, 83]. For example, in dyads, relatives' access to healthcare at baseline was associated with poorer physical QOL among YBCS at follow‐up. Relatedly, in triads, lack of barriers to care was negatively associated with YBCS mental QOL at follow‐up and older relatives' physical QOL at baseline was negatively associated with younger relatives' physical QOL at follow‐up. One potential explanation for these findings could be due to YBCS supporting the care of older relatives who were accessing and utilizing care for their own health issues. While access to care is critical for health and well‐being, as the use of out‐patient services and medical treatments continue to evolve, increased expectations are being placed on family members to provide informal support [84]. Longstanding research has noted that caregiving can have an impact on well‐being, though limited studies take into account the possibility of mutual care within families [85, 86]. Thus, these findings highlight that in addition to recognizing YBCS as care recipients in the context of family survivorship, it is important to recognize YBCS as caregivers and consider the potential affects of caregiving on YBCS's health.

Further interpretation of the partner effects found in our study would benefit from additional qualitative and quantitative research, particularly where results may be considered surprising or unexpected. Overall, however, the findings seem aligned with a family comorbidity perspective [87, 88], which recognizes the connectedness between health issues and concerns of family members, mutual caregiving and support, the strains associated with caregiving, as well as the need to address concurrent family health issues to support and enhance overall well‐being [85]. For example, more than 80% of relatives in our study were first degree relatives to the YBCS and with the majority being sisters. Studies consistently report that family exchanges and concerns evolving around increased cancer risk due to heredity involve primarily first‐degree relatives, as opposed to more distant relatives [89, 90, 91]. The similarities and differences observed in how specific characteristics influence QOL in family members based on their role (YBCS vs. older relatives vs. younger relatives) and type of relationship (e.g., sibling vs. parent/child) also highlights the importance of unpacking the differential ways in which individual members of a family system are affected by chronic illnesses. Future studies should explore whether degree and type of biological relationship has a significant effect on interdependence in QOL or other long‐term cancer‐related outcomes. Furthermore, as this study is one of the first of its kind, future studies should also test the associations investigated in this study to determine if/where results can be replicated. Interventions that target predictors and outcomes for YBCS and relatives have the potential to capitalize on synergistic effects [92, 93].

### 4.2 Limitations

As YBCS were asked to invite relatives to participate in the study, it is likely that they invited relatives with whom they had a supportive relationship and who were in relatively good health. While the inclusion of triads in this study allows for research that goes beyond dyadic relationships, the family units may not be representative of larger family systems of YBCS. The inclusion of additional family members could have provided a more nuanced understanding of family health during cancer survivorship. Consistently, some of the effects noted in the dyads were not present in the triads and vice versa. YBCS who enrolled as dyads may be significantly different compared to those who enrolled as triads; for example, they may have had fewer supportive relationships within their family or smaller family units which could influence perceptions of their own and their family's health‐related outcomes. Exploring the percent of eligible biological female relatives who could have participated in the study with the YBCS and the reasons eligible relatives chose not to participate went beyond the scope of this study; however, this could be valuable to test in future studies. Moreover, while we had several insignificant pathways in our analyses (*p* < 0.05), it is possible that the true effect size is smaller than we were able to detect, which means our conclusion of non‐statistically significant effects would be a Type 2 error [94]. Lastly, while the study oversampled for Black/African American YBCS, sample sizes were not large enough to allow for within group comparisons. Understanding differences between and within diverse groups of patients and their familial systems can be useful for tailoring interventions and highlighting the heterogeneity that exists within these social categories. Using race as a proxy for the social experiences of individuals within a specific category has significant limitations [95].

## 5 Conclusions

This is one of the few studies of long‐term YBCS that includes dyad and triad samples with family members. Findings may reflect the dynamics of health management among families who are past the acute phases of a cancer diagnosis and well beyond the periods of adjustment and transition that occur immediately or shortly after cancer treatment. Given the growing number of YBCS and the importance of family support, addressing physical and mental QOL is crucial. Frameworks such as the family survivorship model and caregiver family experience model provide useful directions to guide analyses and interventions. Indeed, interventions that focus on enhancing supportive resources within broader family systems may be useful for improving both physical and mental QOL of entire cohorts of families affected by chronic illnesses. Future studies should focus on long‐term outcomes of a cancer diagnosis at a young age and the effects on QOL for the individual and their family. Replication of our analyses, particularly with larger samples, can help unpack variation across and within family units (e.g., similarities/differences by type of relationship) and clarify some of the inconsistencies observed when comparing dyad and triad results. Finally, extending APIM analysis to include the broader familial context may help capture and explain some of the synergistic effects observed in interventions that target and address the needs both of the YBCS and their family members/caregivers.

## Author Contributions

**Katrina R. Ellis:** conceptualization (lead), formal analysis (lead), methodology (lead), project administration (lead), writing – original draft (lead), writing – review and editing (lead). **Helen Koechlin:** conceptualization (equal), methodology (equal), writing – original draft (equal), writing – review and editing (equal). **Marion Rudaz:** conceptualization (equal), methodology (supporting), writing – original draft (supporting), writing – review and editing (supporting). **Lynette Hammond Gerido:** conceptualization (supporting), writing – original draft (supporting), writing – review and editing (supporting). **Hillary K. Hecht:** conceptualization (supporting), writing – original draft (supporting), writing – review and editing (supporting). **Carly Jones:** conceptualization (supporting), writing – original draft (supporting), writing – review and editing (supporting). **Dolapo Raji:** conceptualization (supporting), writing – original draft (supporting), writing – review and editing (supporting). **Laurel Northouse:** conceptualization (supporting), writing – original draft (supporting), writing – review and editing (supporting). **Maria Katapodi:** conceptualization (equal), data curation (lead), funding acquisition (lead), investigation (lead), methodology (equal), supervision (lead), writing – original draft (equal), writing – review and editing (equal).

## Ethics Statement

The Institutional Review Board at the University of Michigan approved the study protocol.

## Consent

Written informed consent was obtained from all study participants.

## Conflicts of Interest

The authors declare no conflicts of interest.

## Permission to Reproduce Material From Other Sources

No copyrighted materials or tools were used in this research.

## Supporting information


Table S1.

## Data Availability

The authors have nothing to report.
