# Supplementary material for: Predictors and Interdependence of Quality of Life in a Random Sample of Long‐Term Young Breast Cancer Survivors and Their Biological Relatives
Source: Cancer Med. 2024 Oct 29;13(20):e70328. doi: 10.1002/cam4.70328 (PMC11519995; doi:10.1002/cam4.70328)
Supplement: Supplementary file 1 — Table S1. [file CAM4-13-e70328-s001.docx]

| **Supplemental Table S1.**  **Factors Predicting Physical Quality of Life of YBCS - Dyad** | | | |
| --- | --- | --- | --- |
|  |  |  |  |
| Actor Effects (YBCS --> YBCS Physical QOL) | | |  |
|  |  |  |  |
| **YBCS Factors** | **B** | **S.E.** | **P-Value** |
| Age | 0.302 | 0.114 | 0.008 |
| Education | 0.733 | 0.488 | 0.133 |
| Marital Status | -0.046 | 1.331 | 0.972 |
| Income | -0.052 | 0.368 | 0.887 |
| No cost barriers | 6.082 | 1.447 | <.001 |
| Depression | -1.949 | 1.303 | 0.135 |
| Anxiety | 0.652 | 1.216 | 0.592 |
| Employment | -0.972 | 1.09 | 0.372 |
| Routine source of care | 1.317 | 2.299 | 0.567 |
| Years since diagnosis | -0.362 | 0.158 | 0.022 |
| Physical QOL (baseline) | 0.681 | 0.048 | <.001 |
| Family support | -0.884 | 0.56 | 0.114 |
| Fear of recurrence | 0.078 | 0.084 | 0.353 |
| Perceived breast cancer risk | -0.636 | 0.227 | 0.005 |
|  |  |  |  |
|  |  |  |  |
| Partner Effects (Relative --> YBCS Physical QOL) | | |  |
|  |  |  |  |
| **Relative Factors** | **B** | **S.E.** | **P-Value** |
| No cost barriers | -2.118 | 1.298 | 0.103 |
| Depression | -2.408 | 1.305 | 0.065 |
| Anxiety | -0.496 | 1.43 | 0.729 |
| Routine source of care | -3.913 | 1.868 | 0.036 |
| Physical QOL (baseline) | -0.028 | 0.059 | 0.635 |
| Family support | 0.973 | 0.581 | 0.094 |
| Perceived breast cancer risk | -0.025 | 0.229 | 0.913 |
| Race | -0.999 | 1.307 | 0.445 |

| **Supplemental Table S2.**  **Factors Predicting Physical Quality of Life of Relatives - Dyad** | | | |
| --- | --- | --- | --- |
|  |  |  |  |
| Actor Effects (Relative --> Relative Physical QOL) | | |  |
|  |  |  |  |
| **Relative Factors** | **B** | **S.E.** | **P-Value** |
| Age | -0.05 | 0.039 | 0.198 |
| Education | -0.769 | 0.454 | 0.091 |
| Marital | -1.843 | 0.99 | 0.063 |
| Income | 0.726 | 0.312 | 0.02 |
| No cost barriers | -1.058 | 1.214 | 0.383 |
| Depression | -1.909 | 1.207 | 0.114 |
| Anxiety | 2.519 | 1.24 | 0.042 |
| Employment | 0.43 | 0.941 | 0.647 |
| Routine source of care | 2.512 | 1.463 | 0.086 |
| Physical QOL (baseline) | 0.702 | 0.058 | 0 |
| Family support | 1.474 | 0.544 | 0.007 |
| Perceived breast cancer risk | -0.234 | 0.204 | 0.251 |
|  |  |  |  |
| Partner Effects (YBCS --> Relative Physical QOL) | | |  |
|  |  |  |  |
| **Partner Factors** | **B** | **S.E.** | **P-Value** |
| No cost barriers | -0.169 | 1.121 | 0.88 |
| Depression | 0.289 | 1.202 | 0.81 |
| Anxiety | 0.314 | 1.127 | 0.781 |
| Routine source of care | -2.308 | 1.625 | 0.156 |
| Years since diagnosis | 0.108 | 0.105 | 0.307 |
| Physical QOL (baseline) | 0.047 | 0.042 | 0.268 |
| Family support | 0.577 | 0.471 | 0.221 |
| Fear of recurrence | 0.004 | 0.073 | 0.958 |
| Perceived breast cancer risk | -0.551 | 0.209 | 0.009 |
| Race | -0.117 | 1.184 | 0.921 |

| **Supplemental Table S3.**  **Factors Predicting Physical Quality of Life of YBCS - Triad** | | | | | |
| --- | --- | --- | --- | --- | --- |
|  | |  |  |  | |
| Actor Effects (YBCS --> YBCS Physical QOL) | | | |  | |
|  | |  |  |  | |
| **YBCS Factors** | | **B** | **S.E.** | **P-Value** | |
| Age | | -0.04 | 0.186 | 0.829 | |
| Race | | 3.078 | 2.093 | 0.141 | |
| Education | | -0.868 | 0.988 | 0.38 | |
| Marital status | | 0.385 | 1.721 | 0.823 | |
| Income | | 0.739 | 0.544 | 0.174 | |
| No cost barriers | | 2.186 | 2.255 | 0.332 | |
| Depression | | 3.739 | 1.922 | 0.052 | |
| Anxiety | | -3.099 | 1.869 | 0.097 | |
| Years since diagnosis | | -0.236 | 0.25 | 0.346 | |
| Physical QOL (baseline) | | 0.854 | 0.096 | <.001 | |
| Family support | | 0.045 | 0.958 | 0.963 | |
| Fear of recurrence | | -0.037 | 0.14 | 0.79 | |
| Employment | | 0.194 | 1.44 | 0.893 | |
| Routine source of care | | -1.526 | 3.896 | 0.695 | |
| Perceived breast cancer risk | | -0.285 | 0.362 | 0.432 | |
|  | |  |  |  | |
| Partner Effects (Older Relative --> YBCS Physical QOL) | | | | | |
|  | |  |  |  | |
| **Older Relative Factors** | | **B** | **S.E.** | **P-Value** | |
| Depression | | -2.697 | 2.125 | 0.204 | |
| Anxiety | | 2.409 | 2.054 | 0.241 | |
| Physical QOL (baseline) | | 0.099 | 0.068 | 0.145 | |
| Family support | | -0.061 | 0.935 | 0.948 | |
| Routine source of care | | -0.145 | 3.06 | 0.962 | |
| Perceived breast cancer risk | | 0.449 | 0.405 | 0.267 | |
| No cost barriers | | -0.532 | 2.481 | 0.83 | |
|  | |  |  |  | |
| Partner Effects (Younger Relative --> YBCS Physical QOL) | | | | | |
|  | |  |  |  | |
| **Younger Relative Factors** | | **B** | **S.E.** | **P-Value** | |
| Depression | | 3.433 | 1.779 | 0.054 | |
| Anxiety | | -1.789 | 1.968 | 0.363 | |
| Physical QOL (baseline) | | 0.153 | 0.151 | 0.31 | |
| Family support | | 1.545 | 0.932 | 0.097 | |
| Routine source of care | | 5.048 | 2.051 | 0.014 | |
| Perceived breast cancer risk | | -0.11 | 0.356 | 0.758 | |
| No cost barriers | | 1.074 | 2.177 | 0.622 | |
| **Supplemental Table S4.**  **Factors Predicting Physical Quality of Life of Older Relatives - Triad** | | | | | |
|  | |  |  | |  |
| Actor Effects (Older Relative --> Older Relative Physical QOL) | | | | | |
|  | |  |  | |  |
| **Older Relative Factors** | | **B** | **S.E.** | | **P-Value** |
| Age | | 0.042 | 0.062 | | 0.492 |
| Education | | 0.586 | 0.659 | | 0.374 |
| Marital status | | -2.836 | 1.542 | | 0.066 |
| Income | | 1.205 | 0.433 | | 0.005 |
| No cost barriers | | -0.832 | 1.947 | | 0.669 |
| Depression | | 1.062 | 1.763 | | 0.547 |
| Anxiety | | 0.535 | 1.737 | | 0.758 |
| Physical QOL (baseline) | | 0.76 | 0.061 | | <.001 |
| Family support | | 0.417 | 0.748 | | 0.577 |
| Employment | | -0.151 | 1.308 | | 0.908 |
| Routine source of care | | -1.253 | 2.692 | | 0.642 |
| Perceived breast cancer risk | | 0.133 | 0.307 | | 0.666 |
|  | |  |  | |  |
| Partner Effects (YBCS --> Older Relative Physical QOL) | | | | | |
|  | |  |  | |  |
| **YBCS Factors** | | **B** | **S.E.** | | **P-Value** |
| Depression | | 1.224 | 1.544 | | 0.428 |
| Anxiety | | -0.817 | 1.606 | | 0.611 |
| Physical QOL (baseline) | | 0.206 | 0.077 | | 0.007 |
| Family support | | 1.311 | 0.832 | | 0.115 |
| Routine source of care | | 5.479 | 2.738 | | 0.045 |
| Perceived breast cancer risk | | 0.001 | 0.289 | | 0.996 |
| No cost barriers | | -3.265 | 1.868 | | 0.081 |
| Years since diagnosis | | -0.216 | 0.146 | | 0.139 |
| Fear of recurrence | | -0.075 | 0.117 | | 0.521 |
| Race | | -1.626 | 1.652 | | 0.325 |
|  | |  |  | |  |
| Partner Effects (Younger Relative --> Older Relative Physical QOL) | | | | | |
|  | |  |  | |  |
| **Younger Relative Factors** | | **B** | **S.E.** | | **P-Value** |
| Depression | | -1.329 | 1.483 | | 0.37 |
| Anxiety | | 2.421 | 1.628 | | 0.137 |
| Physical QOL (baseline) | | 0.032 | 0.125 | | 0.799 |
| Family support | | -1.405 | 0.822 | | 0.087 |
| Routine source of care | | 1.314 | 1.704 | | 0.44 |
| Perceived breast cancer risk | | -0.235 | 0.274 | | 0.391 |
| No cost barriers | | 3.074 | 1.786 | | 0.085 |

| **Supplemental Table S5.**  **Factors Predicting Physical Quality of Life of Younger Relatives - Triad** | | | | | | | |
| --- | --- | --- | --- | --- | --- | --- | --- |
|  | | |  | |  | |  |
| Actor Effects (Younger Relative --> Younger Relative Physical QOL) | | | | | | | |
|  | | |  | |  | |  |
| **Younger Relative Factors** | | | **B** | | **S.E.** | | **P-Value** |
| Age | | | -0.142 | | 0.051 | | 0.005 |
| Education | | | -0.464 | | 0.765 | | 0.544 |
| Marital status | | | -2.804 | | 1.227 | | 0.022 |
| Income | | | 1.186 | | 0.363 | | 0.001 |
| No cost barriers | | | -1.293 | | 1.606 | | 0.421 |
| Depression | | | -0.352 | | 1.235 | | 0.776 |
| Anxiety | | | 2.301 | | 1.388 | | 0.097 |
| Physical QOL (baseline) | | | 0.327 | | 0.114 | | 0.004 |
| Family support | | | 0.703 | | 0.635 | | 0.268 |
| Employment | | | 0.225 | | 1.17 | | 0.848 |
| Routine source of care | | | 0.374 | | 1.566 | | 0.811 |
| Perceived breast cancer risk | | | -0.066 | | 0.256 | | 0.797 |
|  | | |  | |  | |  |
| Partner Effects (YBCS --> Younger Relative Physical QOL) | | | | | | | |
|  | | |  | |  | |  |
| **YBCS Factors** | | | **B** | | **S.E.** | | **P-Value** |
| Depression | | | 2.358 | | 1.369 | | 0.085 |
| Anxiety | | | -3.967 | | 1.315 | | 0.003 |
| Physical QOL (baseline) | | | 0.07 | | 0.065 | | 0.281 |
| Family support | | | 0.335 | | 0.694 | | 0.629 |
| Routine source of care | | | -0.146 | | 2.121 | | 0.945 |
| Perceived breast cancer risk | | | 0.012 | | 0.248 | | 0.961 |
| No cost barriers | | | 0.757 | | 1.506 | | 0.615 |
| Years since diagnosis | | | 0.048 | | 0.144 | | 0.739 |
| Fear of recurrence | | | 0.128 | | 0.102 | | 0.206 |
| Race | | | -0.708 | | 1.539 | | 0.645 |
|  | | |  | |  | |  |
| Partner Effects (Older Relative --> Younger Relative Physical QOL) | | | | | | | |
|  | | |  | |  | |  |
| **Older Relative Factors** | | | **B** | | **S.E.** | | **P-Value** |
| Depression | | | -0.637 | | 1.659 | | 0.701 |
| Anxiety | | | 0.579 | | 1.499 | | 0.699 |
| Physical QOL (baseline) | | | -0.121 | | 0.054 | | 0.026 |
| Family support | | | -0.014 | | 0.801 | | 0.986 |
| Routine source of care | | | 1.168 | | 2.287 | | 0.61 |
| Perceived breast cancer risk | | | 0.586 | | 0.282 | | 0.038 |
| No cost barriers | | | 2.005 | | 1.758 | | 0.254 |
| **Supplemental Table S6.**  **Factors Predicting Mental Quality of Life of YBCS - Dyad** | | | | | |  |  |
|  |  |  | |  | |  |  |
| Actor Effects (YBCS --> YBCS Mental QOL) | |  | |  | |  |  |
|  |  |  | |  | |  |  |
| **YBCS Factors** | **B** | **S.E.** | | **P-Value** | |  |  |
| Age | -0.336 | 0.161 | | 0.037 | |  |  |
| Race | 3.087 | 1.749 | | 0.078 | |  |  |
| Education | 0.347 | 0.666 | | 0.602 | |  |  |
| Marital status | 5.579 | 1.824 | | 0.002 | |  |  |
| Income | 0.199 | 0.5 | | 0.69 | |  |  |
| No cost barriers | -3.309 | 1.988 | | 0.096 | |  |  |
| Depression | 0.312 | 1.915 | | 0.871 | |  |  |
| Anxiety | -2.372 | 1.656 | | 0.152 | |  |  |
| Employment | -0.742 | 1.463 | | 0.612 | |  |  |
| Routine source of care | -2.647 | 3.1 | | 0.393 | |  |  |
| Years since diagnosis | 0.148 | 0.217 | | 0.495 | |  |  |
| Mental QOL (baseline) | 0.52 | 0.086 | | <.001 | |  |  |
| Fear of recurrence | -0.186 | 0.114 | | 0.101 | |  |  |
| Perceived breast cancer risk | 0.296 | 0.31 | | 0.341 | |  |  |
| Family support | 0.398 | 0.777 | | 0.609 | |  |  |
|  |  |  | |  | |  |  |
| Partner Effects (Relative --> YBCS Mental QOL) | | | |  | |  |  |
|  |  |  | |  | |  |  |
| **Relative Factors** | **B** | **S.E.** | | **P-Value** | |  |  |
| No cost barriers | 2.525 | 1.773 | | 0.154 | |  |  |
| Depression | 2.105 | 1.758 | | 0.231 | |  |  |
| Anxiety | 0.252 | 1.936 | | 0.896 | |  |  |
| Routine source of care | 0.551 | 2.584 | | 0.831 | |  |  |
| Mental QOL (baseline) | 0.053 | 0.073 | | 0.47 | |  |  |
| Family support | -0.173 | 0.826 | | 0.834 | |  |  |
| Perceived breast cancer risk | 0.258 | 0.324 | | 0.425 | |  |  |

| **Supplemental Table S7.**  **Factors Predicting Mental Quality of Life of Relatives - Dyad** | | | |
| --- | --- | --- | --- |
|  |  |  |  |
| Actor Effects (Relative --> Relative Mental QOL) | | |  |
|  |  |  |  |
| **YBCS Factors** | **B** | **S.E.** | **P-Value** |
| Age | -0.03 | 0.062 | 0.63 |
| Race | 3.438 | 1.898 | 0.07 |
| Education | -0.332 | 0.708 | 0.639 |
| Marital status | 0.262 | 1.613 | 0.871 |
| Income | 0.153 | 0.496 | 0.758 |
| No cost barriers | 0.139 | 1.929 | 0.943 |
| Depression | -1.197 | 1.912 | 0.531 |
| Anxiety | -5.822 | 1.988 | 0.003 |
| Employment | 2.537 | 1.514 | 0.094 |
| Routine source of care | 2.255 | 2.243 | 0.315 |
| Mental QOL (baseline) | 0.426 | 0.074 | 0 |
| Family support | 0.933 | 0.884 | 0.291 |
| Perceived breast cancer risk | 0.035 | 0.315 | 0.912 |
|  |  |  |  |
| Partner Effects (YBCS --> Relative Mental QOL) | | |  |
|  |  |  |  |
| **Relative Factors** | **B** | **S.E.** | **P-Value** |
| No cost barriers | 1.419 | 1.776 | 0.424 |
| Depression | -1.029 | 1.949 | 0.598 |
| Anxiety | 2.629 | 1.745 | 0.132 |
| Routine source of care | -4.284 | 2.58 | 0.097 |
| Mental QOL (baseline) | -0.083 | 0.08 | 0.295 |
| Years since diagnosis | -0.132 | 0.165 | 0.425 |
| Fear of recurrence | 0.043 | 0.115 | 0.711 |
| Perceived breast cancer risk | 0.366 | 0.32 | 0.252 |
| Family support | 0.346 | 0.757 | 0.648 |

| **Supplemental Table S8.**  **Factors Predicting Mental Quality of Life of YBCS - Triad** | | | | | | | | | | | |
| --- | --- | --- | --- | --- | --- | --- | --- | --- | --- | --- | --- |
|  |  | | | |  | | |  | | | |
| Actor Effects (YBCS --> YBCS Mental QOL) | | | | | | | |  | | | |
|  |  | | | |  | | |  | | | |
| **YBCS Factors** | **B** | | | | **S.E.** | | | **P-Value** | | | |
| Age | -0.09 | | | | 0.19 | | | 0.636 | | | |
| Race | -1.456 | | | | 2.248 | | | 0.517 | | | |
| Education | 0.31 | | | | 0.913 | | | 0.735 | | | |
| Marital status | -0.664 | | | | 1.789 | | | 0.711 | | | |
| Income | 0.422 | | | | 0.565 | | | 0.455 | | | |
| No cost barriers | 2.424 | | | | 2.308 | | | 0.293 | | | |
| Depression | -3.469 | | | | 2.174 | | | 0.111 | | | |
| Anxiety | -0.255 | | | | 1.968 | | | 0.897 | | | |
| Years since diagnosis | -0.251 | | | | 0.258 | | | 0.331 | | | |
| Mental QOL (baseline) | 0.438 | | | | 0.096 | | | <.001 | | | |
| Family support | 1.89 | | | | 1.072 | | | 0.078 | | | |
| Fear of recurrence | -0.064 | | | | 0.152 | | | 0.672 | | | |
| Employment | 1.202 | | | | 1.485 | | | 0.419 | | | |
| Routine source of care | 5.069 | | | | 4.024 | | | 0.208 | | | |
| Perceived breast cancer risk | -0.254 | | | | 0.39 | | | 0.516 | | | |
|  |  | | | |  | | |  | | | |
| Partner Effects (Older Relative --> YBCS Mental QOL) | | | | | | | | | | | |
|  |  | | | |  | | |  | | | |
| **Older Relative Factors** | **B** | | | | **S.E.** | | | **P-Value** | | | |
| Depression | 1.697 | | | | 2.109 | | | 0.421 | | | |
| Anxiety | -5.304 | | | | 2.115 | | | 0.012 | | | |
| Mental QOL (baseline) | -0.126 | | | | 0.104 | | | 0.226 | | | |
| Family support | 0.655 | | | | 1.063 | | | 0.538 | | | |
| Routine source of care | -1.415 | | | | 3.284 | | | 0.667 | | | |
| Perceived breast cancer risk | -0.444 | | | | 0.453 | | | 0.327 | | | |
| No cost barriers | -5.319 | | | | 2.667 | | | 0.046 | | | |
|  |  | | | |  | | |  | | | |
| Partner Effects (Younger Relative --> YBCS Mental QOL) | | | | | | | | | | | |
|  |  | | | |  | | |  | | | |
| **Younger Relative Factors** | **B** | | | | **S.E.** | | | **P-Value** | | | |
| Depression | -1.688 | | | | 1.964 | | | 0.39 | | | |
| Anxiety | -1.952 | | | | 2.035 | | | 0.337 | | | |
| Mental QOL (baseline) | -0.037 | | | | 0.117 | | | 0.75 | | | |
| Family support | 0.494 | | | | 1.079 | | | 0.647 | | | |
| Routine source of care | -0.236 | | | | 2.289 | | | 0.918 | | | |
| Perceived breast cancer risk | 0.445 | | | | 0.391 | | | 0.255 | | | |
| No cost barriers | 1.823 | | | | 2.204 | | | 0.408 | | | |
| **Supplemental Table S9.**  **Factors Predicting Mental Quality of Life of Older Relative - Triad** | | | | | | | | | | |  |
|  | | |  | |  | | |  | | |  |
| Actor Effects (Older Relative --> Older Relative Mental QOL) | | | | | | | | | | |  |
|  | | |  | |  | | |  | | |  |
| **Older Relative Factors** | | | **B** | | **S.E.** | | | **P-Value** | | |  |
| Age | | | 0.013 | | 0.063 | | | 0.837 | | |  |
| Education | | | -1.649 | | 0.75 | | | 0.028 | | |  |
| Marital status | | | 2.572 | | 1.677 | | | 0.125 | | |  |
| Income | | | 0.157 | | 0.504 | | | 0.755 | | |  |
| No cost barriers | | | -2.65 | | 2.257 | | | 0.24 | | |  |
| Depression | | | -6.127 | | 1.858 | | | 0.001 | | |  |
| Anxiety | | | -5.536 | | 1.885 | | | 0.003 | | |  |
| Mental QOL (baseline) | | | 0.545 | | 0.087 | | | <.001 | | |  |
| Family support | | | 1.177 | | 0.881 | | | 0.182 | | |  |
| Employment | | | 0.37 | | 1.411 | | | 0.793 | | |  |
| Routine source of care | | | -2.863 | | 2.993 | | | 0.339 | | |  |
| Perceived breast cancer risk | | | -0.406 | | 0.305 | | | 0.183 | | |  |
|  | | |  | |  | | |  | | |  |
| Partner Effects (YBCS --> Older Relative Mental QOL) | | | | | | | | | | |  |
|  | | |  | |  | | |  | | |  |
| **YBCS Factors** | | | **B** | | **S.E.** | | | **P-Value** | | |  |
| Depression | | | -0.453 | | 1.821 | | | 0.803 | | |  |
| Anxiety | | | 1.73 | | 1.741 | | | 0.32 | | |  |
| Mental QOL (baseline) | | | -0.137 | | 0.087 | | | 0.116 | | |  |
| Family support | | | -1.621 | | 0.984 | | | 0.099 | | |  |
| Routine source of care | | | 0.09 | | 2.988 | | | 0.976 | | |  |
| Perceived breast cancer risk | | | -0.043 | | 0.36 | | | 0.905 | | |  |
| No cost barriers | | | 1.712 | | 1.895 | | | 0.366 | | |  |
| Years since diagnosis | | | 0.413 | | 0.162 | | | 0.011 | | |  |
| Fear of recurrence | | | 0.167 | | 0.128 | | | 0.192 | | |  |
| Race | | | 3.94 | | 1.784 | | | 0.027 | | |  |
|  | | |  | |  | | |  | | |  |
| Partner Effects (Younger Relative --> Older Relative Mental QOL) | | | | | | | | | | |  |
|  | | |  | |  | | |  | | |  |
| **Younger Relative Factors** | | | **B** | | **S.E.** | | | **P-Value** | | |  |
| Depression | | | 0.21 | | 1.698 | | | 0.902 | | |  |
| Anxiety | | | 0.807 | | 1.742 | | | 0.643 | | |  |
| Mental QOL (baseline) | | | -0.09 | | 0.095 | | | 0.344 | | |  |
| Family support | | | 0.746 | | 0.966 | | | 0.44 | | |  |
| Routine source of care | | | -0.637 | | 1.996 | | | 0.749 | | |  |
| Perceived breast cancer risk | | | 0.289 | | 0.312 | | | 0.355 | | |  |
| No cost barriers | | | 2.92 | | 1.927 | | | 0.13 | | |  |
| **Supplemental Table S10.**  **Factors Predicting Mental Quality of Life of Younger Relative - Triad** | | | | | | | | | | | |
|  | | | |  | | |  | | |  | |
| Actor Effects (Younger Relative --> Younger Relative Mental QOL) | | | | | | | | | | | |
|  | | | |  | | |  | | |  | |
| **Younger Relative Factors** | | | | **B** | | | **S.E.** | | | **P-Value** | |
| Age | | | | 0.125 | | | 0.079 | | | 0.114 | |
| Education | | | | -0.654 | | | 1.298 | | | 0.614 | |
| Marital status | | | | 1.336 | | | 2.134 | | | 0.531 | |
| Income | | | | -0.32 | | | 0.622 | | | 0.608 | |
| No cost barriers | | | | 2.261 | | | 2.5 | | | 0.366 | |
| Depression | | | | 3.884 | | | 2.198 | | | 0.077 | |
| Anxiety | | | | -1.324 | | | 2.347 | | | 0.573 | |
| Mental QOL (baseline) | | | | 0.674 | | | 0.14 | | | <.001 | |
| Family support | | | | 0.894 | | | 1.192 | | | 0.453 | |
| Employment | | | | -0.09 | | | 2.151 | | | 0.967 | |
| Routine source of care | | | | 0.028 | | | 2.782 | | | 0.992 | |
| Perceived breast cancer risk | | | | 0.108 | | | 0.415 | | | 0.795 | |
|  | | | |  | | |  | | |  | |
| Partner Effects (YBCS --> Younger Relative Mental QOL) | | | | | | | | | | | |
|  | | | |  | | |  | | |  | |
| **YBCS Factors** | | | | **B** | | | **S.E.** | | | **P-Value** | |
| Depression | | | | -3.307 | | | 2.513 | | | 0.188 | |
| Anxiety | | | | 3.041 | | | 2.223 | | | 0.171 | |
| Mental QOL (baseline) | | | | 0.044 | | | 0.116 | | | 0.707 | |
| Family support | | | | 1.137 | | | 1.294 | | | 0.38 | |
| Routine source of care | | | | 4.194 | | | 3.549 | | | 0.237 | |
| Perceived breast cancer risk | | | | -0.262 | | | 0.496 | | | 0.597 | |
| No cost barriers | | | | -1.037 | | | 2.454 | | | 0.673 | |
| Years since diagnosis | | | | 0.297 | | | 0.255 | | | 0.243 | |
| Fear of recurrence | | | | -0.359 | | | 0.177 | | | 0.043 | |
| Race | | | | 0.619 | | | 2.382 | | | 0.795 | |
|  | | | |  | | |  | | |  | |
| Partner Effects (Older Relative --> Younger Relative Mental QOL) | | | | | | | | | | | |
|  | | | |  | | |  | | |  | |
| **Older Relative Factors** | | | | **B** | | | **S.E.** | | | **P-Value** | |
| Depression | | | | -4.116 | | | 2.781 | | | 0.139 | |
| Anxiety | | | | 4.789 | | | 2.528 | | | 0.058 | |
| Mental QOL (baseline) | | | | -0.091 | | | 0.114 | | | 0.421 | |
| Family support | | | | 0.562 | | | 1.328 | | | 0.672 | |
| Routine source of care | | | | -2.843 | | | 3.898 | | | 0.466 | |
| Perceived breast cancer risk | | | | 0.505 | | | 0.446 | | | 0.257 | |
| No cost barriers | | | | -3.205 | | | 2.877 | | | 0.265 | |
